# Supplementary material for: Training Methods Used by Dog Guardians in the United States: Prevalence, Sources of Information, and Reasons for Use
Source: Animals (Basel). 2024 Apr 27;14(9):1310. doi: 10.3390/ani14091310 (PMC11083790; doi:10.3390/ani14091310)
Supplement: Supplementary file 1 [file animals-14-01310-s001.zip › animals-2965301-supplementary.pdf]

Survey questions presented to student participants

Human Demographic information	
Q1: Gender	Male<br>Female<br>Other<br>Choose not to answer
Q2: Age	18-30<br>31-40<br>41-50<br>51-60<br>61-70<br>71-80<br>80+

Q3: Income Bracket (Please provide the income range for the home where the dog resides).	0-10k<br>11-30k<br>31-50k<br>51-70k<br>71-90k<br>90-110k<br>120-250k<br>250k+
Q4 Zip Code	
Q5: How many individuals (including yourself) live in your household?	
Q6: Other individuals in the household: Select as many options as you need to cover all members of your household.	Partner/Spouse<br>Children under 10<br>Children 11-18<br>Older children<br>Other relative<br>Other adult
Q7: Please choose one word that best describes your relationship to your dog	Parent<br>Owner<br>Guardian<br>Friend<br>Master/Mistress<br>Family member (e.g. sister, brother, sibling) *(Added for Fall 2021 survey)*<br>Other
Dog Demographic information: Please answer for the dog you spend most time with. If you are not able to distinguish among your dogs, answer questions for the dog whose name comes first in the alphabet.	
Q8: Dog's Date of Birth (write unknown if date is not known)	

Q9: Dog's Current Age: Please write in years, allowing for decimal e.g.: .5 year (6 months)	
Q10: Gender	Male, intact<br>Female, intact<br>Male, neutered<br>Female, spayed
Q11: Where did you first hear about the dog?	Friend/Family Member<br>Internet (Rescue site, Petfinder, Craigslist, internet search)<br>Printed Media<br>Visit to physical location (e.g.: breeder, shelter, pet store) without specific animal in mind<br>Other
Q13: Where did you end up getting the dog?	Breeder<br>Pet Store<br>Bred at home<br>Rescue (municipal shelter, private shelter, foster-based rescue)<br>Friend/Family member<br>Internet<br>Other
Training Methodology	
Q14: I most regularly walk my dog on a …	Flat Collar<br>Martingale<br>Chain Collar<br>Prong Collar<br>Back-clip Harness<br>Front-clip Harness<br>Head Halter<br>I do not walk my dog

Q15: While inside the house, my dog wears (select all that apply)	Collar<br>No collar (naked)<br>Harness<br>Activity monitor<br>Citronella Collar<br>Electronic Collar (to manage barking or other behavior)<br>Electronic Collar (for invisible fence system)
Q16: Training Class Experience	My dog and I have attended at least one training class/consult<br>I have attended training meetings (Enter the number of class sessions your dog attended)<br>I am presently taking my dog to training meetings<br>I have utilized a private trainer (Enter the number of sessions your dog attended)<br>I am presently utilizing a private trainer<br>I trained my dog myself<br>My dog has not received any formal training
If participant selected that the dog did not receive any formal training—	
Q17: If you noted you did not receive any formal training, please select why<br>*Participants then directed to Q21*	Because my dog is perfect<br>Because my dog was previously trained<br>Because classes are too expensive<br>Didn’t have time<br>Other
If participant selected that attended any training ---	
Q18: How old was your dog when you first attended training class (class or with private trainer)<br>Please write in years, allowing for decimal e.g.: .5 year (6 months)	

Q19: How old was your dog when you last attended training class (class or with private trainer<br>Please write in years, allowing for decimal e.g.: .5 year (6 months)	
Q20: If you sought training with a trainer, how would you describe their methods.	Use of rewards, treats, petting, praise, clickers, no corrections<br>Use of corrections only (e.g.: electronic collar, spray bottle, tapping with item, alpha roll etc.)<br>Mixed use of rewards and corrections
Q22: When I have concerns about my dog's behavior, I will ask for help from...	My veterinarian<br>A friend or family member<br>Research and try techniques from online (websites, YouTube)<br>Research and try techniques that I have seen on social media (e.g.: Facebook, Instagram, TikTok) *(Option added for Fall 2021)*<br>Directly ask a dog trainer<br>The breeder or shelter/rescue where I got my dog from
Q23: I have learned how to do deal with my dog's behavior from:<br>Please select the book that you reference the most	Monks of New Skete: e.g.: The Art of Raising a Puppy<br>Cesar Millan: e.g.: How to raise the Perfect Dog<br>Sophia Yin: e.g.: How to behave so your dog behaves<br>Zac George: e.g.: Dog Training Revolution<br>Brandon McMillan: e.g.: Lucky Dog Lessons<br>Other<br>None

Q24: I have learned how to do deal with my dog's behavior from:<br>Please select the television show that you reference the most	The Dog Whisperer (Cesar Millan)<br>It’s Me or the Dog (Victoria Stillwell)<br>Dog Impossible (Matt Beisner)<br>Canine Intervention (Jas Leverette)<br>Lucky Dog (Brandon McMillian)<br>Other<br>None
Q25: I have learned how to do deal with my dog's behavior from:<br>Please select the method of social media that you reference the most<br>*(Option added for Fall 2021)*	Facebook<br>Instagram<br>TikTok<br>Twitter
If selected any social media option:<br>Please list some people or groups that you follow for behavior advice	
Managing Behavior	
Q26: Pulls on Leash	Auditory Correction (person makes loud noise, use of physical item to make noise (Pet corrector, shake can etc.)<br>Physical Correction (pinch, push, etc.)<br>Reward alternative behavior<br>Management of dog’s access to whatever might be bothering dog<br>Use of electronic collar (human or bark activated)<br>Specialized Leash equipment (prong collar, harness, choke chain)<br>Ignore<br>Time Outs<br>This is not a problem my dog has

Q27: Barking in the home (e.g.: when alone, at activity outside of the house, demand barking)	Auditory Correction (person makes loud noise, use of physical item to make noise (Pet corrector, shake can etc.)<br>Physical Correction (pinch, push, etc.)<br>Reward alternative behavior<br>Management of dog's access to whatever might be bothering dog<br>Use of electronic collar (human or bark activated)<br>Ignore<br>Time Outs<br>This is not a problem my dog has
Q28: Aggression to dog (e.g.: barking, lunging, growling, biting)	Auditory Correction (person makes loud noise, use of physical item to make noise (Pet corrector, shake can etc.)<br>Physical Correction (pinch, push, etc.)<br>Reward alternative behavior<br>Management of dog's access to whatever might be bothering dog<br>Use of electronic collar (human or bark activated)<br>Ignore<br>Time Outs<br>This is not a problem my dog has

Q29: Aggressive to people (e.g.: bark, lunge, growl, bite)	Auditory Correction (person makes loud noise, use of physical item to make noise (Pet corrector, shake can etc.)<br>Physical Correction (pinch, push, etc.)<br>Reward alternative behavior<br>Management of dog's access to whatever might be bothering dog<br>Use of electronic collar (human or bark activated)<br>Ignore<br>Time Outs<br>This is not a problem my dog has

Q30: Destructive Behavior (e.g.: destroys inappropriate items like clothing, furniture etc.)	Auditory Correction (person makes loud noise, use of physical item to make noise (Pet corrector, shake can etc.)<br>Physical Correction (pinch, push, etc.)<br>Reward alternative behavior<br>Management of dog’s access to whatever might be bothering dog<br>Use of electronic collar (human or bark activated)<br>Ignore<br>Time Outs<br>This is not a problem my dog has
Q31: Jumping up on people	Auditory Correction (person makes loud noise, use of physical item to make noise (Pet corrector, shake can etc.)<br>Physical Correction (pinch, push, etc.)<br>Reward alternative behavior<br>Management of dog’s access to whatever might be bothering dog<br>Use of electronic collar (human or bark activated)<br>Ignore<br>Time Outs<br>This is not a problem my dog has

Q32: Resource Guarding (e.g.: barking, lunging, growling when dog is either by food or items)	Auditory Correction (person makes loud noise, use of physical item to make noise (Pet corrector, shake can etc.)<br>Physical Correction (pinch, push, etc.)<br>Reward alternative behavior<br>Management of dog's access to whatever might be bothering dog<br>Use of electronic collar (human or bark activated)<br>Ignore<br>Time Outs<br>This is not a problem my dog has
Q33: Does not respond when called (e.g.: will not come back when off leash either in home or outside)	Auditory Correction (person makes loud noise, use of physical item to make noise (Pet corrector, shake can etc.)<br>Physical Correction (pinch, push, etc.)<br>Reward alternative behavior<br>Management of dog's access to whatever might be bothering dog<br>Use of electronic collar (human or bark activated)<br>Ignore<br>Time Outs<br>This is not a problem my dog has
If participant selected any management strategy for any of the problem behaviors	

Q34-Q41: Please select what led you to utilize this method for leash pulling	Recommendation from a trainer<br>Recommendation from a friend/family member<br>Recommendation from a vet<br>Saw this method online (websites, YouTube)<br>Saw this method on social media (Facebook, Instagram, TikTok, Twitter) *(Option added for Fall 2021)*<br>Saw this method in a book<br>Saw this method on a TV show<br>Other
Participant reported efficacy	
Q42: Of all the training techniques you have used with your dog, what technique do you think has been LEAST effective?	Use of rewards, treats, petting, praise, clickers, no corrections<br>Use of corrections only (e.g.: electronic collar, spray bottle, tapping with item, alpha roll etc.)<br>Mixed use of rewards and corrections
Q43: Of all the training techniques you have used with your dog, what technique do you think has been MOST effective?	Use of rewards, treats, petting, praise, clickers, no corrections<br>Use of corrections only (e.g.: electronic collar, spray bottle, tapping with item, alpha roll etc.)<br>Mixed use of rewards and corrections
Q44: Why do you think this technique was effective? Please select no more than two options	Dog does not show the behavior anymore<br>Ease of Use<br>Better ability to control the dog<br>Tolerance by the dog<br>Inexpensive<br>Worked well with my dog's learning style/motivation
